# Dental Stem Cell-Derived Exosomes: A Review of Their Isolation, Classification, Functions, and Mechanisms

**DOI:** 10.1155/2024/2187392

**Published:** 2024-08-16

**Authors:** Xiner Ning, Rui Liu, Yingying Huang, Zhilong Huang, Haodi Li, Qiqi Li, Zengyan Sheng, Junjie Wu

**Affiliations:** ^1^ Department of Orthodontics School of Stomatology State Key Laboratory of Military Stomatology and National Clinical Research Center for Oral Diseases and Shaanxi Clinical Research Center for Oral Diseases The Fourth Military Medical University, Xi'an 710032, China; ^2^ State Key Laboratory of Oral and Maxillofacial Reconstruction and Regeneration National Clinical Research Center for Oral Diseases Shaanxi Key Laboratory of Stomatology Nursing Department School of Stomatology The Fourth Military Medical University, Xi'an 710032, China

## Abstract

The scientific field concerned with the study of regeneration has developed rapidly in recent years. Stem cell therapy is a highly promising therapeutic modality for repairing tissue defects; however, several limitations exist, such as cytotoxicity, potential immune rejection, and ethical issues. Exosomes secreted by stem cells are cell-specific secreted vesicles that play a regulatory role in many biological functions in the human body; they not only have a series of functional roles of stem cells and exert the expected therapeutic effects, but they can also overcome the mass limitations of stem cells and are thus considered in the research as an alternative treatment strategy for stem cells. Since dental stem cell-derived exosomes (DSC-Exos) are easy to acquire and present modulating effects in several fields, including neurovascular regeneration and craniofacial soft and hard tissue regeneration processes, they are served as an emerging cell-free therapeutic strategy in various systematic diseases. There is a growing body of research on various types of DSC-Exos; however, they lack systematic elaboration and tabular summarization. Therefore, this review presents the isolation, characterization, and phenotypes of DSC-Exos and focuses on their current status of functions and mechanisms, as well as the multiple challenges prior to clinical applications.

## 1. Introduction

Exosomes were first identified in the year 1986 from the supernatant of sheep erythrocytes [1]. They are small, single-membrane, secreted organelles with a diameter of 30–200 nm that possess the same topology as cells; they are enriched in selected proteins, lipids, nucleic acids, and glycoconjugates [2, 3], and they exist in high quantities in body fluids [4]. There are two forms of exosomal biogenesis: one is the endosome-dependent budding pathway, and the other is the direct budding pathway of the plasma membrane [3, 5]. The former pathway is regarded in the literature as the main generation pathway that promotes the processes of exosome germination and release, depending on the mechanism of the endosomal sorting complex required for transportation (ESCRT) [6]. ESCRT-Ⅰ and ESCRT-Ⅱ initiate the germination process on the outer surface of endosomal membranes, forming intraluminal vesicles and multicysts, which participate in the protein deubiquitination process through ESCRT-Ⅲ, drive vesicle separation [7], and fuze with the plasma membrane to form exosomes [3]. Exosomes are selectively transported to adjacent or distant cells present in the extracellular matrix. They play an important role in mediating cellular communication, signal transduction, antigen presentation, and the epigenetic reprograming of receptor cells by means of the direct stimulation of receptor cells through cell surface ligands, the transportation of functional proteins, and the delivery of RNAs and transcription factors to the receptor cell, which, in turn, regulate human functions [8, 9, 10, 11, 12].

Mesenchymal stem cells (MSCs) are multipotent stem cells with the ability to self-renew and differentiate in multiple directions. MSCs-derived exosomes, produced by paracrine mechanisms of MSCs, are applied as a source of acellular therapy due to low acquisition cost, efficient function, long-term storage, and high recovery rates [3, 13]. Compared to MSCs, MSCs-derived exosomes can effectively overcome the drawbacks of cell therapy, such as cytotoxicity, immune rejection, difficulty in regulation, and low precision [12]. The tissue sources of MSCs are usually bone marrow, umbilical cord, adipose tissue, and oral tissue. Dental stem cell-derived exosomes (DSC-Exos), compared with exosomes derived from other sources of MSCs such as bone marrow mesenchymal stem cells (BMMSCs) derived exosomes, are easily accessible and less traumatic, and possess fewer ethical issues, since they are collected from oral tissue [14]. Thus, this review focuses on the status of various researches on DSC-Exos in the field.

## 2. DSC-Exos

### 2.1. Classification

**Figure 1:**
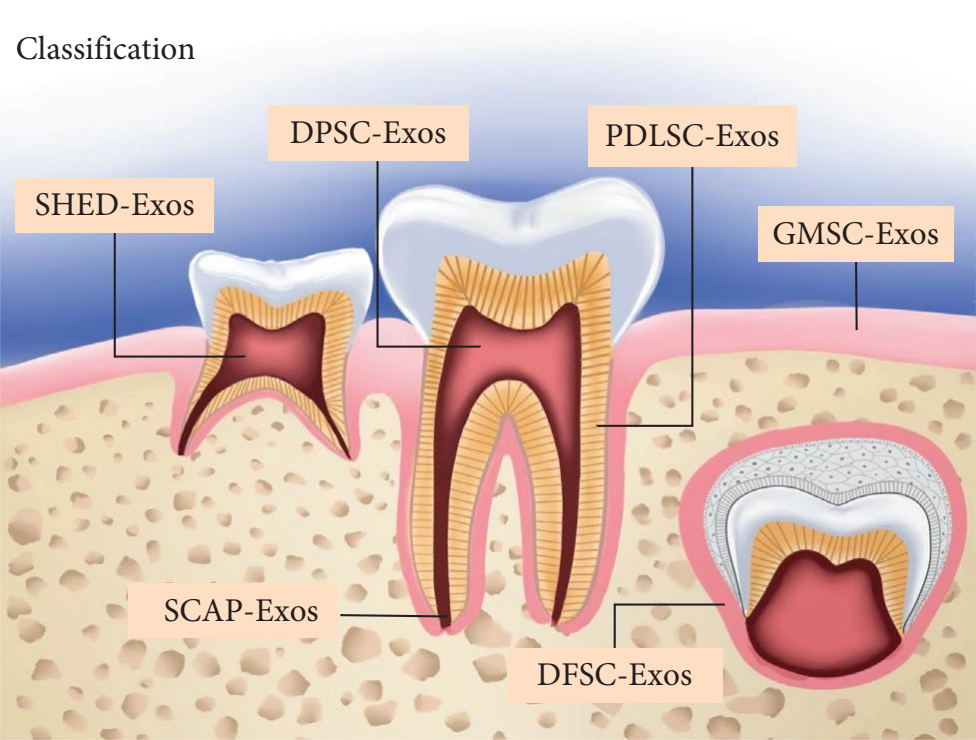
Classification of DSC-Exos.

According to the most recent literature presented by Mai et al. [15], DSC-Exos can be divided into periodontal ligament stem cell-derived exosomes (PDLSC-Exos), dental pulp stem cell-derived exosomes (DPSC-Exos), stem cells obtained from human exfoliated deciduous teeth-derived exosomes (SHED-Exos), gingival mesenchymal stem cell-derived exosomes (GMSC-Exos), stem cells from apical papilla-derived exosomes (SCAP-Exos), and dental follicle stem cell-derived exosomes (DFSC-Exos) [16], which is relevant in various fields, such as osteogenesis, anti-inflammation, tissue regeneration, tumor suppression, and the treatment of neurodegenerative diseases (Figure 1).

Referred to as the seed cell for periodontal regeneration purposes, PDLSCs have proved to be a reliable source of exosomes, and they present substantive potential applications in a clinical setting [17]. The exosomes of PDLSCs possess an extremely high application potential in osteogenesis [18], anti-inflammation [19], angiogenesis [20], and periodontal regeneration processes [17]. DPSCs were isolated and cultured from the pulp of a human third molar by Gronthos et al. [21] through enzymatic digestion. They are renowned for their multidifferentiation, self-renewal, and high proliferation abilities [22]. DPSCs can migrate to the damaged tissue area, secrete a variety of functional factors, and support the regeneration of damaged tissue, among which exosomes participate in the interaction of paracrine between cells and play a therapeutic role by inducing the endogenous repair process. SHEDs were the first heterogeneous stem cell group isolated from deciduous incisors by Miura et al. [23]. They are rich in growth factors and present considerable advantages in their pluripotency and proliferation abilities [5]; therefore, SHEDs and their exosomes have a great application potential in various fields. GMSCs are divided into two subpopulations, neural crest outer mesenchymal origin (N-GMSC) and mesodermal origin (M-GMSC), with the former being better differentiated into neuronal cells [24]. Gingival tissue is considered a good source from which to derive stem cells due to its minimally invasive procedure and rapid regeneration capability following an injury. GMSC-Exos are involved in numerous processes. They contain many growth factors and participate in the differentiation and angiogenesis of osteoblasts. The glial cell-derived neurotrophic factor family ligands and neurotrophic factors are involved in their processes of neuronal development, anti-inflammatory, and tissue regeneration [25]. SCAPs were first isolated from the developing papilla of young permanent teeth by Sonoyama et al. [26]. They were inoculated with a tricalcium hydroxyapatite phosphonate complex culture for a period of 4 hr and then transplanted to the dorsal subcutis of nude mice, and the formation of pulp–dentin-like structures was observed histologically after 8 weeks [27]. SCAP-Exos were also shown to be an ideal stem cell source for pulp–dentin complex and soft tissue regeneration processes [28]. DFSCs derived from ectomesenchyme participate in the development and eruption of teeth and form periodontal tissues. However, relatively few studies on the exosomes of DFSC-Exos have been conducted in recent years, which mainly involve periodontitis treatment and periodontal tissue regeneration [29].

### 2.2. Isolation

Currently, there is no standardized protocol for isolating exosomes [30]. Methods, such as differential ultracentrifugation [31, 32, 33], ultrafiltration [34], immunoaffinity [35], and the nonspecific precipitation method [36], are commonly used in the research. The above-mentioned isolation methods are also applicable to DSC-Exos, among which differential ultracentrifugation is adopted the most [31]. The typical ultracentrifugation protocol includes successive centrifugations at increasing speeds: low-speed (10^2^x *g* and 10^3^x *g*) centrifugation to pellet any contaminating cell and eliminate dead cells, followed by higher-speed centrifugation (10^4^x *g*) to remove cell debris [37]. At each of these steps, the pellet is thrown away, and the supernatant is used for the following step [38]. Then, the filtered supernatant was ultracentrifuged (10^5^x *g*) for once or twice, and the purified exosomes were resuspended in PBS and used for further examination [18]. Intriguingly, we counted 40 studies and found that very few of the centrifugal forces and timings of each procedure applied in different literatures are exactly the same (Table 1), even for centrifuges of the same brand. Besides, exosome extraction and isolation kits designed according to the membrane structure characteristics of exosomes are also welcomed in recent years [58, 76, 77, 78, 79, 80, 81, 82, 83].

### 2.3. Characterization

DSC-Exos are verified using flow cytometry to assess the phenotypic characteristics. Western Blot was employed to detect expression-specific exosome markers, including tetraspanins (CD9, CD63, CD81) and ESCRT-associated components (Alix and tumor susceptibility gene 101, TSG101) [59, 74, 75, 84, 85, 86] (Table 2). The total exosome concentration was quantified using microbicinchoninic acid (BCA) Protein Assay Kit [65, 72], and the morphology and particle size were usually assessed by the transmission electron microscopy and the nanoparticle tracking analysis [72, 73, 74]. In addition, atomic force microscopy could provide information on both surface morphology and material properties (stiffness, adhesion) by amplitude modulation and phase modulation [97].

### 2.4. Functions

**Figure 2:**
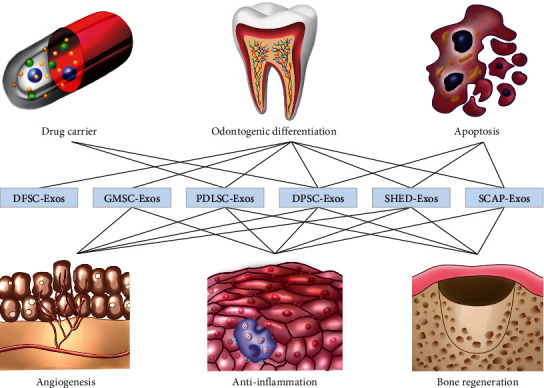
Pathophysiological functions of phenotypes of DSC-Exos.

Since DSC-Exos present modulating effects in several fields of research, such as neurovascular regeneration and craniofacial soft and hard tissue regeneration processes, the research conducted on them at present is dramatically increasing [98] (Figure 2).

One of the most essential features of DSC-Exos is their potential to promote odontogenic differentiation and regeneration activity. DPSC-Exos were observed in the research to regenerate the pulp–dentin complex, mainly by mimicking the microenvironment associated with dentin development [47, 91], and promote the deposition of calcium and collagen fibers [99, 100], while SCAP-Exo was also found to promote the formation of dentin salivary glandular phosphoproteins and mineralized nodule formations, as well as regenerate pulpal dentin-like tissue [101, 102]. PDLSC-Exos and SHED-Exos were also observed to promote stem cell odontogenic differentiation activity [76, 90].

Most DSC-Exos have proangiogenic effects, among which SHED-Exos and GMSC-Exos present dual regulatory effects, inhibiting the expression of oxidative stress-induced proangiogenic factors and reducing the occurrence of tumor micro-angiogenesis [28, 77, 103]; meanwhile, these exosomes also upregulate the proangiogenic VEGF-related pathway and promote skin-wound healing activity [65, 95].

In response to the bone loss associated with periodontitis, odontogenic exosomes play a vital role in regulating the inflammatory microenvironment and inducing osteogenesis. A variety of odontogenic exosomes can reduce inflammatory responses through immunomodulation, specifically by inhibiting the activity of histone proteases and matrix metalloproteinases (MMPs) at the site of inflammation [60, 61], thus affecting the polarization of macrophages [69, 70, 72, 73, 88], suppressing the production of inflammatory factors [19, 39, 45, 70, 104], affecting Th17/Treg homeostasis [28, 38, 45], and suppressing NF-*κ*B and TLR4 pathways [39, 71, 88], thus improving the microenvironment. As for the osteogenic properties, studies conducted in the literature on the effect of PDLSC-Exos on osteogenic potential are priorities for researchers at present. PDLSC-Exos also present a dual regulatory effect on osteogenesis: on the one hand, they promote the processes of proliferation, migration, and the osteogenic differentiation of MSCs, as well as the regeneration of the alveolar bone in patients exhibiting acute periodontitis in response to bone loss [18, 40, 43, 84, 105]; on the other hand, they can also promote bone reconstruction behavior, resulting in the inhibition of osteogenic differentiation under PGE2 induction [42, 87]. DPSC-Exos, SHED-Exos, and SCAP-Exos can also promote osteogenic differentiation behavior, among which SCAP-Exos can present a high osteogenic induction ability following inoculation on 3D PLA scaffolds [82, 96].

SHED-Exos and GMSC-Exos exhibit highly neurologically relevant induction properties that modulate a variety of neurological diseases, such as Parkinson's and TBI [62, 66, 79]. The former can promote various outcomes, neuronal axon growth, microglia glycolytic reprograming, and polarization toward the anti-inflammatory M2 phenotype, and thus inhibit neuronal inflammation [63, 66, 79]; they can also promote the occurrence of neuronal apoptosis [58]. Moreover, it can also affect the normalized expression of tyrosine hydroxylase, improving motor symptoms [62].

Three types of DSC-Exos, including DPSC-Exos, SHED-Exos, and SCAP-Exos, reduced the risk of apoptosis by regulating the expression of apoptosis-related proteins. Furthermore, DPSC-Exos and SCAP-Exos reduced the risk of apoptosis [28, 106], while SHED-Exos promoted apoptosis occurrence in vascular endothelial cells and dopaminergic neurons [57].

In addition, PDLSC-Exos and DPSC-Exos could inhibit tumor cell proliferation by way of the drug carrier [41, 49], and GMSC-Exos were shown to induce tastebud regeneration [107].

### 2.5. Mechanisms

**Figure 3:**
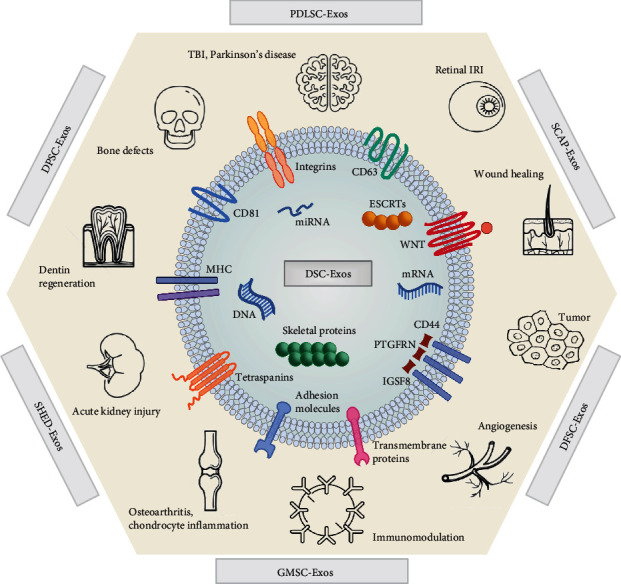
Mechanisms of phenotypes of DSC-Exos.

DSC-Exos express rich RNA profiles [25], mainly including messenger RNA (mRNA) [108], microRNA (miRNA) [109], PIWI-interacting RNA (piRNA) [102], long noncoding RNA (lncRNA) [110], and circular noncoding RNA (circRNA) [44]. These RNA, especially miRNA, exert as indispensable regulators of exosome functions. The functions and their mechanisms of six types of DSC-Exos are described in detail below (Figure 3 and Table 3).

#### 2.5.1. PDLSC-Exos

PDLSC-Exos regulate the osteogenic differentiation of cells in the human organism in both *in vivo* and *in vitro* settings, thus promoting the expression of osteogenic-related proteins (including Osterix and boney-containing protein, BGP) and forming mineralized nodules [113]. For instance, the exosomes of inflammatory periodontal ligament stem cells extracted from periodontitis tissues (i-PDLSCs) following gallic acid induction could remarkably promote the osteo-differentiation of i-PDLSCs [43]. This ability is realized by regulating adenosine receptor signaling pathways, such as Wnt, phosphoinositide 3-kinase (PI3K/Akt), and mitogen-activated protein kinase (MAPK) signaling pathways. PDLSC-Exos can also inhibit the expression of an essential molecule downstream of the over-activated Wnt signaling pathway: *β*-Catenin [105]. ERK plays a crucial role in the tertiary kinase cascade reaction of the MAPK signaling pathway; its phosphorylation is a key mediator during enhanced BMMSCs migration activity. The infusion of PDLSC-Exos increases the number of exosomal protein annexin A3 (ANXA3) to facilitate the exosome internalization process, which activates ERK and inhibits H_2_O_2_-induced apoptosis, which activates the PI3K/AKT and MEK/ERK signaling pathways, thus inducing osteoclast differentiation [18, 84, 87]. Meanwhile, PDLSC-Exos present a bidirectional regulating effect on the osteogenic differentiation process; its overexpression of miR-34c-5p inhibits osteogenesis via targeting special AT-rich sequence-binding protein 2 and reducing the phosphorylation of ERK1/2.

Studies have shown that PDLSC-Exos' regulatory effects on the osteogenic differentiation process, such as the adenosine receptor signaling pathway described above, are closely related to the expression level of their RNAs. RNA sequencing showed that exosomes contain a variety of noncoding RNAs, including antisense RNAs, long-stranded noncoding RNAs, and miRNAs, among which Chiricosta et al. [114] highlighted in their research the presence of noncoding RNAs and five miRNAs, including miR24-2, miR142, miR335, miR490, and miR29, which target the genes classified in two gene ontology categories: “Ras protein signal transduction” and “Actin/microtubule cytoskeleton organization”. The RNA expression profiles of the exosomes were significantly altered following the osteogenic differentiation, and 3 circRNAs, 2 lncRNAs, and 72 miRNAs were upregulated, and 39 circRNAs, 5 lncRNAs, and 35 miRNAs were downregulated [16, 44], and when the stem cells were modified in the P2X7R gene, miR-3679-5p, miR-6515-5p, and miR-6747-5p were also highly expressed [104]. These differentially expressed RNA exomes were observed to be enriched in pathways, such as the MAPK signaling pathway, thereby enhancing the osteogenic capacity of PDLSCs [40].

PDLSC-Exos are expected by the researchers to solve the problem of alveolar bone resorption behavior in patients with chronic periodontitis based on their excellent ability to promote osteogenic differentiation and regulate immune responses to play an additional anti-inflammatory role. In their study, Pizzicannella et al. [82] evidenced the activation of bone regeneration and vascularization processes by rats implanted with 3D-PLA/hGMSCs/EVs. The vascularization of periodontal ligaments was mediated by the VEGF-VEGFR signaling pathway and the nuclear factor kappa-light-chain-enhancer of the activated nuclear factor kappa-light-chain-enhancer of activated B cell (NF-*κ*B) signaling pathway. PDLSC-Exos were observed to modulate miR-17-5p, targeting the former pathway [20], and mediate the PI3K/Akt signaling pathway suppressing the latter [39, 115]. PDLSC-Exos also mediate paracrine effects to improve the inflammatory micro-environment where the Gremlin 1 protein of the TGF-*β*/BMP signaling pathway plays a central role. MiR-3679-5p and miR-6747-5p were highly expressed and bound directly to the Gremlin 1 protein [104]. Moreover, miR-155-5p was highly transferred into CD4^+^ T cells to further regulate the expression of the silent mating-type information regulation 2 homolog-1 (SIRT1), thereby affecting the balance of T helper cells (Th17)/regulatory cells (Treg) in the inflammatory microenvironment [38].

The miRNAs enriched in PDLSC-Exos discovered in the research were not only observed to be powerful but also diverse, and some of them are also associated with proto-oncogenes, which indicates that exosomes also have certain therapeutic effects on tumors [114]. In their research, Fei et al. [41] observed that PDLSC-Exos regulate intercolonial communication among squamous cell carcinomas with osteogenic heterogeneity through the upregulation of PINK1/parkin-mediated mitophagy, which further affected the proliferation and differentiation processes of target cells.

#### 2.5.2. DPSC-Exos

DPSC-Exos also present great application prospects in the field of pulp–dentin complex regeneration. The transforming growth factor *β*-1/drosophila mothers against the decapentaplegic protein (Smads) pathway triggered the odontogenic differentiation of DPSC lineage and induced the formation of pulp-dentin-like neurovascular tissues [90, 116]. Given that DPSC-Exos possess promising regenerative properties, scholars have constructed a number of novel modalities suitable for clinical settings. In their study, Swanson et al. [48] exploited both mineralizing primary human dental pulp stem cells and an immortalized murine odontoblast cell line MDPC-23 to design an amphiphilic synthetic polymeric vehicle from a triblock copolymer, which allowed for the encapsulation and controlled, tunable release of cell-derived exosomes, and modulated downstream recipient cells towards a designed dentinogenic trajectory in both *in vitro* and *in vivo* settings. Chen et al. [47] placed SCAP-containing collagen gel on the root tip and filled the cavity of the treated dental matrix with DPT-Exo and DPC-Exo-laden scaffolds, which would be expected to recruit SCAPs to the pulp cavity and then regenerate dental pulp-like connective tissues containing collagen, odontoblasts, and enriched predentin-like tissue. Furthermore, Guo et al. [91] established a strategy using a decellularized tooth matrix combined with human dental pulp stem cell aggregates containing DPSC-Exos to simulate an odontogenesis-related developmental microenvironment by implanting reconstructed bioengineered teeth into an alveolar bone. Moreover, they enrolled 15 patients, implanted the bioengineered teeth, and realized the regeneration of functional teeth 12 months later [91]. Moreover, the overexpression of calcium sensor protein stromal interaction molecule 1 (STIM1) promoted the release of DPSC-Exos and the mineralized matrix, further affecting the dentin mineralization process [100].

DPSC-Exos can also address the problem of alveolar bone resorption in periodontitis well and modulate the inflammatory immune microenvironment. DPSC-Exos were also observed to stimulate the migration of human DPCs and osteoblastic cells [89] and coincidentally increased the expression of circular lysophosphatidic acid receptor 1 (circLPAR1) to eliminate the inhibitory effect of hsa-miR-31 on osteogenesis [56]. In terms of the regulation of immunomodulation, DPSC-Exos showed stronger immunomodulatory activity than BMMSCs-Exos. They can induce the transition of CD4+ cell differentiation from Th17 to Treg and decrease the secretions of pro-inflammatory factors IL-17 and TNF-*α*, while releasing anti-inflammatory factors IL-10 and TGF-*β* [45]. They mediated miR-1246 expression to facilitate the macrophages converting from pro-inflammatory to anti-inflammatory phenotypes [50, 88].

In addition to promoting hard tissue regeneration, DPSC-Exos also have good therapeutic potential for the soft tissue regeneration process [53]. Zhang et al. [54] co-cultured endothelial cells and DPSCs in EV-fibrin gels and a vascular-like structure generated by increasing the release of VEGF and the deposition of collagen-type I, III, and IV. Exosomes secreted by DPSCs isolated from periodontally compromised teeth or under hypoxia-preconditioning conditions led to higher expression levels of angiogenesis-related genes/proteins and a quicker healing outcome than those secreted from periodontally healthy ones [46, 93].

Based on its ability to cross the blood–brain barrier, DPSC-Exos can also be applied to neurological-related diseases [117]. LPS-preconditioned DPSC-Exos have the capacity to regulate Schwann cell proliferation, migration, and odontogenic differentiation processes [55]. They present neuroprotective efficacy, can be used to treat spinal cord injury (SCI) by reducing macrophage M1 polarization [52], alleviate the damage of cerebral ischemia-reperfusion injury (IRI) by mediating the HMGB1/TLR4/MyD88/NF-*κ*B signaling pathway [51], and repair hippocampal neuron degeneration by activating the cell survival PI3K-B-cell lymphoma-2 (Bcl-2) pathway [85].

Moreover, DPSC-Exos can also be used as drug carriers to suppress tumor growth activity, such as glioblastomas and breast carcinomas [49, 106], and inhibit the occurrence of chondrocyte apoptosis [57, 97].

#### 2.5.3. SHED-Exos

Under different pretreatment conditions, SHED-Exos have a bidirectional induction effect on the process of angiogenesis. In an ectopic tooth model implanted subcutaneously in the backs of mice, SHED-Exos shuttled miR-26a and mediated the TGF-*β*/SMAD2/3 signaling pathway, contributing to the occurrences of angiogenesis and endothelial differentiation [67]. LPS-stimulated SHED-Exos altered 10 types of miRNA expressions to promote angiogenesis and also mediated the transfer processes of miR-100-5p and miR-1246 to induce the apoptosis of vascular endothelial cells [65, 80]. Hypoxic preconditioned SHED-Exos significantly reduced microangiogenesis occurrence in xenografted OSCC tumors by transferring let-7f-5p and miR-210-3p [77].

Strikingly, SHEDs have a therapeutic potential for a variety of neurological disorders due to their neural crest origin. In a traumatic brain injury (TBI) rat model, SHED-Exos inhibited neuronal inflammation and promoted neuronal axon growth through the expression of miR-124-3p [66]. In a TBI rat model, SHED-Exos could reduce neuroinflammation outcomes by shifting microglia polarization and improved rat motor functional recovery outcomes [79]. In response to the neurological effects of 6-hydroxydopamine, SHED-Exos were able to inhibit 6-hydroxydopamine-induced apoptosis in dopaminergic neurons and also normalized the expression levels of tyrosine hydroxylase present in the substantia nigra and striatum [58, 62].

In terms of inducing the osteogenic differentiation of cells, SHED-Exos can act on various types of MSCs. SHED-Exos were shown to increase the expression of mitochondrial transcription factor A (TFAM) in DPSCs by transferring TFAM mRNA [111] and, when bound to matrix proteins, such as type-I collagen and fibronectin, they can be endocytosed by DPSCs in a dose-dependent and saturable manner and trigger the P38 MAPK pathway, further enhancing bone metabolism activity [76]. It was observed that SHED-Exos specifically promoted BMMSCs osteogenesis and inhibited lipogenesis based on the upregulation of the expression levels of osteogenic factors Runx2 and p-Smad5 and the reduction in the expression levels of lipogenic markers PPAR*γ* and lipid droplets [64, 68]. Moreover, SHED-Exos also increased the migration, proliferation, and osteogenic differentiation processes of PDLSCs, promoted the cell cycle transition from G1 to S phases, and enhanced Runx2 expression and mineralization [78], and Wang et al. [59] showed that BMP/Smad signaling and Wnt/*β*-catenin were activated by enhanced Smad1/5/8 phosphorylation and increased nuclear *β*-catenin protein expression, which further promoted the osteogenic differentiation of PDLSCs.

In the field of anti-inflammatory research, similar to the previous two exosomes, SHED-Exos were also proven to abrogate inflammatory responses. However, the research conducted on this behavior tends to focus more on the therapeutic role concerning other forms of inflammation than on periodontitis. For instance, SHED-Exos can repress the chondrocyte inflammation of the temporomandibular joint by delivering factors, such as miR-100-5p targeted mTOR [61], significantly inhibits carrageenan-induced acute inflammation in mice by inhibiting the activity of tissue proteinase B and MMPs at the site of inflammation [60], attenuate the inflammatory response in rat pulpitis by upregulating Treg [101], and suppress the LPS-induced activation of the NF-*κ*B signaling pathway in human microglial cells by inducing the significant upregulation of phagocytic activity occurring in M0 cells [63].

#### 2.5.4. GMSC-Exos

GMSC-Exos promote the migration of preosteoblasts and the osteogenic differentiation of MC3T3-E1, thus aiding the formation of bone structure and remodeling the alveolar bone [74]. The increased expression levels of osteogenic and angiogenic markers, such as RUNX2, VEGFA, OPN, and COL1A1, in living construct 3D-PLA/GMSCs/exosomes evidenced the activation of bone regeneration and vascularization processes [82]. Diomede et al. [96] showed improved osteogenic properties located at the injury site following the implantation of a 3D PLA scaffold, GMSCs compounded with GMSC-Exos, into the cranial cortical bone tissues of rats with injuries. In their study, Shi et al. [95] combined GMSC-Exos with hydrogel to promote collagen re-epithelialization and remodeling, angiogenesis, and neurite ingrowth activities, which effectively improved skin-wound healing outcomes in diabetic rats. In addition, GMSC-Exos significantly reduced skin and vascular dysfunctions associated with attenuation and aging processes by eliminating oxidative stress-induced gene expression levels [103]. The study proved that GMSC-Exos reduced the inflammatory immune response of periodontitis by regulating the Wnt5a-mediated RANKL pathway [71], and pretreatment of GMSC-Exos with TNF-*α* could upregulate miR-1260b to further inhibit Wnt5a, thereby contributing to the resolution of inflammation [69].

The anti-inflammatory ability of GMSC-Exos is reflected in their ability to inhibit lipid accumulation in a high-lipid microenvironment, reduce the release of inflammatory factors, and promote the conversion of macrophages into an anti-inflammatory phenotype [72, 73]. Kou et al. [70] showed that this was the result of the activation of the pro-inflammatory cytokines TNF-*α* and IFN-*α* by GMSC-Exos, mediating the Fas/Fap-1/Cav-1 axis that regulates SNARE-mediated exosomes and IL-1RA secretion in stem cells, which contributes to accelerated wound healing results.

Moreover, GMSC-Exos can also be applied to nerve as well as bud regeneration processes. GMSC-Exos can promote peripheral nerve regeneration activity by activating the c-Jun N-terminal kinase-regulated repair phenotype of Schwann cells [94] and can significantly increase the number and diameter of nerve fibers and promote myelin formation following a combination with biodegradable chitin conduits [81]. In their research, Yu et al. [112] suggested that GMSC-Exos can also be used as a cell-free therapeutic approach for glaucoma, and pretreatment with TNF-*α* substantially enhanced their neuroprotective effects on retinal ischemia-reperfusion injury. Moreover, Zhang et al. [107] observed that GMSC-Exos increased the expression levels of CK14 and regenerated-type I, II, and III tastebud cells and further promoted the innervation of regenerated tastebuds.

#### 2.5.5. SCAP-Exos

In the field of tissue regeneration, whether analyzed *in vivo* or *in vitro*, SCAP-Exos promoted pulp–dentin complex formation. SCAP-Exod were introduced into the root fragment containing BMMSCs and transplanted subcutaneously into immunodeficient mice. Dentin was evident in the root fragment [28], and the gene and protein expression levels of dentin sialo phosphoprotein and mineralized nodule formation were significantly increased [118]. This result may be related to the differential expression of piRNA in the exosomes. Wang et al. [102] observed that the 21 differentially expressed piRNAs were mainly involved in biological regulation, cellular processes, metabolic processes, and binding and catalytic activities, which are closely related to the biological functions of MSCs that are closely bound to the dentin regeneration process. In another study, SCAP-Exos also promoted soft tissue regeneration and angiogenesis via delivering exosomal Cdc42 [119].

It is fascinating to note that SCAP-Exos can also be implemented to ameliorate cisplatin-induced nephrotoxicity by inhibiting oxidative stress, inflammatory response, and apoptosis behaviors. This may be achieved by suppressing the signaling pathways, such as sirtuin 1 (SITR1), MAPK, p53, and reactive oxygen species (ROS) [75].

#### 2.5.6. DFSC-Exos

DFSC-Exos may promote PDLSCs to proliferation, migration, osteogenic differentiation, and periodontal tissue regeneration activities by activating the p38 MAPK signaling pathway [29]. Pretreatment with LPS can further improve the therapeutic effects of DFSC-Exos on periodontitis [83], enabling DFSC-Exos to highly express proteins mainly involved in antioxidant and enzyme-regulating activities and acting as an antioxidant to inhibit ROS/JNK signaling and promote macrophages to polarize toward the M2 phenotype via ROS/ERK signaling activity. Furthermore, LPS-preconditioned DFSC-Exos loaded with the HA injectable system could sustainably release exosomes and enhance the therapeutic efficacy for periodontitis in canines [92].

## 3. Perspectives, Challenges, and Solutions

As an emerging cell-free treatment modality, DSC-Exos have shown great therapeutic potential in reducing alveolar bone loss caused by periodontitis, promoting angiogenesis to repair tissue defects, regulating macrophage transformation to reduce inflammatory responses, and inducing neuronal cell proliferation and apoptosis to guide nerve regeneration processes. Thus, the regulatory mechanisms of exosomes are in urgent need of systematic elucidation in the literature. Researchers in the field have explored various regulatory pathways, such as adenosine signaling pathways mediated by miRNAs transported by exosomes, and studied the mechanisms of action of different dental-derived exosomes, slowly unveiling the mechanisms of exosome action; however, the specific and systematic mechanisms still need to be studied and elucidated in the research.

Some studies have shown that the physiological or pathological conditions of the tissue or cell of origin at the time of exosome secretion [120]; the preconditioning stimuli, such as TNF-*α*, hypoxia, LPS, and mechanical strain induction; the intracellular calcium content [121] of secreted exosomes; and the microenvironmental conditions, such as whether the exosomes bind materials, including titin ducts, chitosan hydrogels, *β*-tricalcium phosphate, and fibrin gels, can substantially affect the formation and functional role of exosomes. When DPSC-Exos were applied in the experiments to fibrin-based regenerative root-filling materials, the fibrin gels promoted exosome attraction to MSCs and further promoted the proliferation of bone marrow MSCs [99]. In view of this, in the future, we can focus on the design and development of the exosomal culture microenvironment and loading materials and use related biomaterials in conjunction with the target direction of the action of exosomes to enhance their therapeutic effects.

To date, most studies conducted on the therapeutic application of exosomes remain in the preclinical stage, i.e., the animal experimental stage, and studies performed at the clinical experimental level have yet to be implemented in the research. The specific clinical application mode of exosomes needs to be further explored and standardized in the literature, and its effectiveness and safety outcomes on the human body need to be further evaluated. Some exosomes have been proven to display a senescence phenotype, and as the number of cell culture passages increases, senescent cell derivatives also exhibit a senescence-related secretory phenotype [122]. In addition, the different separation methods are closely related to the purity and yield outcomes of exosomes. Therefore, it is necessary to explore the standardized procedures for exosome isolation and long-term effective storage to study exosome mass production and manufacturing technologies, to develop the application strategy of exosomes with clinical benefits, and to ultimately establish a unified international standard for exosome application, production specification, and quality control methods [68].

Furthermore, in terms of product safety, there is a risk of the co-isolation of endogenous viruses by exosomes during the isolation process, based on the similarity between the physical properties between viruses and exosomes and the fact that the downstream processing steps of exosomes are also more similar to those used for viral vaccine production. Both being essentially composed of functional genetic materials and surface proteins, chemical inactivation may cause as much damage to exosomes as to viruses, thereby destroying functional surface proteins. Finding a way to maximize exosome function while adequately removing the virus is also necessary to ensure exosome biosafety.

Ultimately, although many difficulties still exist in the research and must be resolved before dental-derived exosomes can be clinically applied, their broad clinical application prospects are worthy of further research and development.

## 4. Conclusions

All types of DSC-Exos present considerable advantages and characteristics in the treatment of diseases and conditions in the oral field and are also promising for the treatment of other types of systemic diseases, such as oncology diseases and Parkinson's diseases. Prior to their clinical application, it remains necessary to further evaluate the safety outcomes and standardize the clinical production and application modes of these exomes so that they can be utilized in a real clinical setting as soon as possible.

## Figures and Tables

**Table 1:** Ultracentrifugation settings for isolation of DSC-Exos.

Classification	10^2^ × *g*	Min	10^3^ × *g*	Min	10^4^ × *g*	Min	10^5^ × *g*	Min	Reference
PDLSC-Exos	300	10	2,000	10	20,000	30	100,000	70,70	[39]
	300	5	3,000	15	10,000	70	100,000	70,70	[18]
	300	10	2,000	20	10,000	30	100,000	70	[40]
	300	10	2,000	10	16,000	30	100,000	70,70	[41]
	800	10	—	—	10,000	30	130,000	70,70	[42]
	300	10	—	—	10,000	10	100,000	70,70	[43]
	500	10	—	—	16,000	30	150,000	70	[20]
	—	—	3,000	20	16,500	20	100,000	70	[44]

DPSC-Exos	300	10	—	—	16,500	30	100,000	70,70	[45]
	300	10	2,000	10	10,000	30	100,000	60,60	[46]
	2,000	20	15,000	40	5,000	30	10,000	60	[47]
	300	10	2,000	10	4,000	10	100,000	70,70	[48]
	300	10	2,000	20	10,000	30	100,000	70	[49]
	300	10	—	—	16,500	20	120,000	150	[50]
	300	10	2,000	10	20,000	30	100000	70,70	[51]
	300	10	2,000	10	10,000	30	100,000	70	[52]
	500	10	2,000	30	—	—	100,000	60	[53]
	300	10	2,000	20	10,000	40	110,000	90,90	[54]
	500	10	2,000	10	10,000	60	100,000	120,70	[55]
	—	—	3,000	20	16,500	20	100,000	70	[56]
	—	—	3,000	30	10,000	30	64,000	110	[57]

SHED-Exos	300	10	2,000	10	20,000	30	100,000	70,70	[58, 59, 60, 61, 62, 63, 64]
	300	10	2,000	10	10,000	30	100,000	70,70	[65]
	300	10	2,000	10	10,000	30	100,000	70	[66]
	—	—	2,000	10	10,000	30	100,000	70,70	[67]
	400	5	2,000	15	10,000	30	100,000-	90	[68]

GMSC-Exos	300	10	3,000	10	20,000	30	120,000	70	[69, 70]
	300	10	2,000	20	10,000	30	100,000	70	[71]
	300	10	3,000	20	10,000	30	100,000	70	[72, 73]
	300	10	2,000	10	10,000	30	100,000	70,70	[74]

SCAP-Exos	—	—	3,000	20	20,000	30	120,000	120	[28]
	—	—	2,000	10	12,000	30	100,000	70,70	[75]

**Table 2:** Exosome markers for identification of DSC-Exos.

Classification	Exosome markers	Reference
PDLSC-Exos	CD9, CD63, glyceraldehyde-3-phosphate dehydrogenase (GADPH)	[38]
	CD63, TSG101, calnexin	[40]
	CD9, CD63, CD81, tumor susceptibility 101 (TSG101)	[84]
	CD9, CD63, CD81, TSG101, GADPH	[18]
	CD63, CD81	[44, 87]
	CD9, CD63, CD81, TSG101, PTEN-induced putative kinase 1 (PINK1), Parkin, microtubule-associated protein light chain 3 (LC3-I/II)	[41]
	CD9, CD81, ALIX, *β*-actin	[43]
	CD9, GADPH, E-cadherin, N-cadherin, vimentin, forkhead box protein P3 (FOXP3), retinoic acid-related orphan receptor C (RORC)	[38]
	CD63, TSG101, calnexin	[40]
	CD9, CD81, calnexin, TSG101	[42]
	CD63, VEGF, TSG101, GADPH, *β*-tubulin	[20]
	CD63, CD81, Alix	[64]

DPSC-Exos	CD63, CD81, TSG101, Alix, heat shock 70 kDa protein (HSP70)	[85]
	CD63, CD81	[56]
	CD9, CD63, CD81	[46, 86]
	CD9, CD63	[47, 52, 88]
	CD9, CD81	[89]
	CD9, CD63, Alix, Golgi matrix protein 130 (GM130)	[55, 90]
	CD9, CD63, TSG101	[91]
	CD63, HSP70, TSG101	[92]
	CD9, CD63, TSG101, calnexin	[59]
	CD9, CD63, Bcl-2, rabbit anti-human Bax and Bad	[57]
	CD63, TSG101, calnexin	[49]
	CD9, TSG101, HSP70	[50]
	CD63, TSG-101, calnexin, GAPDH	[54]
	CD9, CD81, Alix, HSP70	[93]

SHED-Exos	CD63, TSG101	[77, 80]
	CD9, CD63	[79]
	Syntenin 1, HSP70, milk fat globule-epidermal growth factor 8 (MFG-E8)	[63]
	CD9, CD63, HSP70	[65, 66]
	CD9, CD63, TSG101, calnexin	[59, 61, 78]
	CD9, CD63, CD81, TSG101	[67]
	CD63, GM130	[64]

GMSC-Exos	CD9, CD81, TSG101, Alix	[71]
	CD9, CD63, CD81, HSP70	[73]
	CD9, CD63, TSG101	[74]
	CD9, CD81, CD63	[72]
	CD9, CD63	[81, 94]
	CD9, CD81	[95]
	CD9, CD63, CD81, TSG101, Runx2, BMP2/4, *β*-actin	[96]

SCAP-Exos	CD9, Ailx	[28, 75]

DFSC-Exos	CD81, TSG101, HSP90	[29]
	CD63, TSG101, HSP70	[92]
	CD63, TSG101, actin, periostin, OPN, OCN, Runx2, GAPDH	[83]

**Table 3:** Functions and mechanisms of DSC-Exos.

Classification	Functions	Mechanism	Exosome processing	Model	Reference
PDLSC-Exos	Bone remodeling and regeneration	Alleviate oxidative stress in inflammatory PDLSCs and increase mitochondrial membrane potential and glycometabolism levels	Gallic acid (GA) pretreatment of stem cell sources	*In vitro*	[43]
		Inhibition of excessive activation of Wnt signaling and expression of its downstream molecule *β*-linked protein	Binding matrix gel or *β*-tricalcium phosphate	*In vitro*: PDLSCs in periodontitis patients with inflammatory periodontal membrane separation *In vivo*: bone defects in a rat model of periodontitis	[105]
		PGE2-induced targeting of special AT-rich sequence-binding protein 2 by overexpression of miR-34c-5p reduces phosphorylation levels of ERK1/2 and inhibits osteogenic differentiation	PGE2-induced	*In vitro*	[42]
		Increases the expression level of annexin A3 induced by mechanical force, activates ERK phosphorylation, and promotes osteoclast differentiation	Binding soluble receptor activator RANKL, mechanically induced	*In vitro*: RAW264.7 macrophages *In vivo*: an animal model of mechanically induced tooth movement	[87]
	Osteogenic differentiation	The expression of 72 miRNAs was upregulated, and 35 miRNAs were downregulated prior to and following osteogenesis-induced differentiation of BMMSCs, and the differentially expressed miRNAs were involved in MAPK, AMPK, and insulin signaling pathways	None	*In vitro*: osteogenic induction medium, conditioned medium	[40]
		Activation of PI3K/AKT and MEK/ERK signaling pathways promotes proliferation, migration, and osteogenic differentiation of hFOB1.19 cells	None	*In vitro*: H_2_O_2_-induced apoptotic cells hFOB1.19	[84]
		Increase in AKT and ERK1/2 enhances the proliferation and migration of bone marrow mesenchymal stem cells	Fixed in Matrigel, combined with ECM holder	*In vivo*: bilateral cranial defect model in rats	[18]
	Inflammatory immunomodulation	Inhibition of inflammatory factor IL-1*β* production in macrophages	None	*In vitro*: anti-inflammatory experiment of chondrocytes, synoviocytes, and meniscus cells	[19]
		Increasing the phosphorylation level of Akt and its downstream target GSK3*β*. Inhibits NF-*κ*B activity and perhaps interferes with the TLR4 signaling pathway	Stem cells derived from gelatin-coated alginate microcarriers	*In vitro*: comparison of basal and LPS-induced inflammatory activities	[39]
		CD4^+^ T cells take up miR-155-5p and regulate the expression of silent-mating-type information regulation 2 homolog-1 protein in target cells, thereby affecting Th17/Treg homeostasis	LPS-stimulated stem cell source	*In vitro*: *Porphyromonas gingivalis* and LPS mimic the inflammatory microenvironment of chronic periodontitis	[38]
		High expression levels of miR-3679-5p, miR-6515-5p, and miR-6747-5p, binding to gremlin 1 protein	P2X7R gene-modified stem cell source	*In vitro*: osteogenic induction medium	[104]
	Angiogenesis	Promotes upregulation of vascular-specific markers CD31 and VEGFA expression, which is regulated as a target of miR-17-5p	None	*In vitro*: lipogenic and osteogenic media, HUVECs cell culture	[20]
	Inhibition of tumor proliferation	Mediated PINK1/parkin-dependent mitochondrial phagocytosis inhibits communication between heterogeneous single-cell clones in squamous cell carcinomas	Treatment with or without exocytogenesis/release inhibitor GW4869	*In vitro*: conditioned medium for high and low osteogenic capacities	[41]

DPSC-Exos	Osteogenic differentiation	Stimulated migration of human periodontal cells and mouse osteoblasts	None	*In vitro* *In vivo*: a mouse model of ligature-induced periodontitis	[89]
		Abolishing the inhibitory effect of hsa-miR-31 on osteogenesis, promoting circLPAR1 expression, and osteogenic differentiation of receptor homotypic DPSCs	Osteogenic-induced stem cell source	In vitro	[56]
	Inflammatory immunomodulation	Possible targeting of miR-1246 promotes the conversion of macrophages from pro-inflammatory phenotype M1 to anti-inflammatory phenotype M2 in periodontal tissues of mice with periodontitis	Bound chitosan hydrogel	*In vitro*	[50]
		Inhibits the differentiation of CD4^+^ T cells to Th17, reduces the release of pro-inflammatory factors IL-17 and TNF-*α*, induces the polarization of CD4^+^ T cells to Treg, and promotes the release of anti-inflammatory factors IL-10 and TGF-*β*	None	*In vitro*: DPSC-Exos and BMMSCs-Exos were co-cultured with peripheral blood mononuclear cells	[45]
		Exosomal miR-125a-3p switched macrophages toward the M2 phenotype via inhibiting NF-*κ*B and TLR signaling via direct IKBKB targeting	None	*In vitro* *In vivo*: dental pulp capping model	[88]
	Angiogenesis	The expression of 79 proteins was significantly altered after hypoxic pretreatment, with Lysyl oxidase homolog 2 expression upregulated	Hypoxia-treated stem cell sources	*In vitro*: exosomes were isolated from DPSCs under normal-treated stem cell-derived exosome and hypoxia-treated stem cell-derived exosome conditions and added to HUVECs	[46]
		Elevated expression levels of angiogenesis-related genes/proteins in ECs	Healthy or inflammatory stem cell sources	*In vitro*	[93]
		Mediated Cdc42/p38 MAPK pathway promotes endothelial cell angiogenesis	None	*In vitro* *In vivo*: 1× 1 cm full-thickness excised skin wound on the back of 8-week-old female mice, subcutaneously injected with exo	[53]
	Pulp regeneration	Unidentified. Regenerated pulp-like connective tissue containing collagen, adult dentin cell progenitors, and enriched anterior dentin-like tissue	None	*In vivo*: collagen gel containing SCAP was placed at the root tip, and the treated dental matrix cavity was filled with a DPC-Exo scaffold to recruit SCAP. *In vitro*: abovementioned complexes were implanted subcutaneously in immunodeficient nude mice, and tissue samples were selected after 8 weeks	[47]
		Unknown. Attracted bone marrow mesenchymal stem cells; fibrin gel enhanced the effect	Binding fiber gel	*In vitro*	[54]
	Dentin generation	Unknown. Designing amphiphilic synthetic polymer-carriers and regulating downstream receptor cells toward designed odontogenesis trajectories	Human dental pulp stem cells derived from primary mineralization and immortalized MDPC-23	*In vitro*: construction of amphiphilic synthetic polymeric carriers based on triblock copolymers *In vivo*: abovementioned vectors were transplanted into a rat pulpotomy model	[48]
		STIM1 promotes calcium deposition, leading to exosome and mineralized matrix release	None	*In vivo*: STIM1-deficient mouse model	[100]
	Regeneration of functionally dislocated teeth	Unknown. Regeneration of three-dimensional pulp and periodontal groups with neurovascular innervations using decellularized dental matrix combined with exosomes to mimic the developmental microenvironment associated with odontogenesis	None	*In vitro*: construction of cellular dental matrix combined with human DPSC-Exos for bioengineered teeth *In vivo*: bioengineered tooth transplantation into 15 patients with avulsed teeth following dental trauma	[91]
	Regenerative root canal treatment	Increases VEGF release and promotes type-I, -III, and -IV collagen depositions	In situ formation of delivery systems for exo-fibrin gel composites	*In vitro*: co-culture of endothelial cells and DPSCs in exo-fibronectin gels	[99]
	Odontogenic differentiation	Stimulation of dentin salivary protein production and mineralization by Schwann cells	Pretreatment with or without LPS	*In vitro*	[55]
		Significant changes in 28 miRNAs in exosomes with a high expression level of MiR-27a-5p promote the odontogenic differentiation of DPSC by downregulating the repressor molecule LTBP1 and upregulating transforming growth factor *β*-1, TGFR1, p-Smad2/3, and Smad4	Separation of hypo-differentiation and odontogenic differentiation conditions	*In vitro*	[90]
	SCI	Targeting the ROS-MAPK-NF-*κ*B P65 signaling pathway to inhibit macrophage M1 polarization	None	*In vitro*: H2O2 pretreatment RAW264.7 cells	[52]
	Inhibits the growth of glioblastoma	Conversion of nontoxic 5-fluorocytosine to the cytotoxic drug 5-fluorouracil	yCD::UPRT-MSC-mediated	*In vivo*: C6 glioblastoma of rat brain	[106]
	Cerebral IRI	Significantly inhibited IRI-mediated expression levels of TLR4, myeloiddifferentiationfactor88 and NF-*κ*B, and protein expression levels of IL-6, IL-1*β*, and TNF-*α*, while suppressing the IRI-induced cytoplasmic translocation of HMGB1	None	*In vitro*: oxygen–glucose deprivation–reperfusion-induced BV2 cells *In vivo*: C57BL/6 mice underwent a 2-hr transient middle cerebral artery occlusion injury, were reperfused for 2 hr, and injected with DPSC-Exos via the tail vein alone	[51]
	Diseases related to hippocampal neuron degeneration	Activation of pi3k-Bcl-2 pathway and upregulation of host endogenous growth factor expression level	None	*In vitro*: red alginate-treated hippocampal cells	[85]
	Osteoarthritis	MiR-140-5-enriched exosomes inhibit chondrocyte apoptosis by regulating the expression level of apoptosis-related proteins and promote the expression of chondrocyte-associated mRNAs, including aggregated glycan, Col2*α*1, and Sox9	MiR-140-5 transfected stem cell source	*In vitro*: human chondrocytes treated with IL-1*β*	[57]
	Drug carrier	Delivery of tumor suppressor miR-34a inhibits breast cancer cell proliferation	Preparation of DPSCs overexpressing miR-34a by XMIRXpress-34a slow vector	*In vitro*	[49]

SHED-Exos	Bone remodeling and regeneration	Different groups containing multiple growth factors, including TGF-*β*1, platelet-derived growth factor, insulin-like growth factor-1, and fibroblast growth factor-2, mobilize initial bone marrow mesenchymal stem cells	Different incubation times (24, 48, and 72 hr)	*In vitro*	[68]
		Promotion of bone marrow MSC osteogenesis, inhibition of lipogenesis, upregulation of Runx2 and p-Smad5, key factors of osteogenic differentiation, and reduction in the expression levels of lipogenic markers PPAR*γ* and lipid droplet volume	None	*In vivo*: a mouse model of ligature-induced periodontitis	[64]
		Promotes the cell cycle transition from G1 to S phases in PDLSCs and enhances their Runx2 expression and mineralization	None	*In vitro*	[78]
		Carrying Wnt3a and BMP2, BMP/Smad signaling pathway and Wnt/*β*-linked proteins are activated by enhanced Smad1/5/8 phosphorylation and increased expression of nuclear *β*-linked proteins	None	*In vitro*	[59]
		Transfer of TFAM mRNA increases mitochondrial transcription factor-A expression and enhances glutamate metabolism and OXPHOS activity in DPSCs	None	*In vivo*: mouse cranial defect model	[111]
	Antiangiogenesis	Downregulation of multiple angiogenesis-related factors, including VEGFA, MMP-9, and ANGPT1, mediated the *in vivo* transfer of miR-100-5p and miR-1246 to induce apoptosis in vascular endothelial cells, thereby significantly reducing tumor microvessel formation generated by xenograft OSCC cells	None	*In vitro* *In vivo*: chicken chorioallantois membrane test and oral squamous cell carcinoma xenograft model	[80]
	Angiogenesis	Elevated mRNA expression levels of VEGF and kinase insertion domain receptor; 10 miRNAs change targeting angiogenesis-related pathways	LPS-stimulated stem cell source	*In vitro*: co-culture with HUVECs	[65]
	Inhibition of microangiogenesis in tumors produced by xenografted OSCC cells	Regulation of angiogenesis via let-7f-5p/AGO1/VEGF and/or miR-210-3p/ephrinA3 signaling pathways under hypoxic conditions and may be associated with Rab27a	Hypoxia preconditioning or normal-culture stem cell source	*In vitro*: endothelial cell assay *In vivo*: matrix gel plug-test model	[77]
	Pulp regeneration	DPSC and HMSC endocytose exosomes delivered in a dose-dependent and saturable manner via a luminal vesicle endocytosis mechanism and triggered the P38 MAPK pathway and increased expression of genes required for odontogenic differentiation	Binds to matrix proteins, such as type-I collagen and fibronectin, and fills the root canal lumen of human molars	*In vitro* *In vivo*: root-section model, implanted subcutaneously in the back of thymus-free nude mice	[76]
		Mediates TGF-*β*/SMAD2/3 signaling pathway and promotes angiogenesis	None	*In vitro*: SHEDs and HUVECs	[67]
	TBI	Altering microglia polarization to reduce neuroinflammation, improve motor recovery, and reduce cortical damage in rats	None	*In vitro*: co-culture with activated BV-2 microglia *In vivo*: TBI rat model	[66]
		Inhibition of PDE4B gene expression by mediating miR-124-3p further inhibits mTOR signaling pathway activity, promotes neuronal axon growth, and the anti-inflammatory M2 phenotype polarization of microglia, thereby suppressing neuronal inflammation	None	*In vivo*: TBI mouse model; collect brain extracts from acute to chronic phases of acute TBI injury	[79]
	Parkinson's disease	Normalization of tyrosine hydroxylase expression level in the substantia nigra and striatum of 6-hydroxydopamine-treated rats, resulting in improvement of motor symptoms in rats	None	*In vivo*: intranasal administration; unilateral 6-hydroxydopamine medial forebrain bundle induced Parkinson's disease model in rats	[62]
	Apoptosis of dopaminergic neurons	Unknown. SHED-derived exosomes grown on laminin-coated three-dimensional alginate microcarriers inhibit 6-hydroxydopamine-induced apoptosis of dopaminergic neurons	Stem cell sources cultured in standard two-dimensional culture flasks or bioreactors in laminin-coated microcarriers	*In vitro*	[58]
	Chondrocyte inflammation of the TMJ	Inhibits the expression levels of IL-6, IL-8, MMP1, MMP3, MMP9, MMP13, and thrombospondin and metalloproteinase 5, and delivers miR-100-5p targeting the untranslated region of mTOR3 to suppress mTOR expression	None	Treatment of chondrocytes with miR-100-5p mimics or rapamycin	[61]
	Carrageenan-induced acute inflammation	Inhibits the activity of tissue proteinase B and MMPs at the site of inflammation and progressively exerts inhibitory effects	None	*In vivo*: carrageenan induction for a plantar injection model of inflammatory ALB/c mice	[60]
	Neuroinflammatory microglia	Inhibition of the LPS-induced NF-*κ*B signaling pathway in human microglia induces altered phagocytic activity in differentially polarized macrophages and glycolytic reprograming in unpolarized and polarized human microglia	None	*In vitro*: lipopolysaccharide-induced human microglia	[63]

GMSC-Exos	Bone remodeling and regeneration	Unknown. Promotion of migration and osteogenic differentiation of preosteoblastic MC3T3-E1	None	*In vitro*	[74]
		RUNX2, VEGFA, OPN, and COL1A1 osteogenesis-related proteins and miR-2861 and miR-210 expression levels are significantly upregulated	Bonded polylactic acid (3D-PLA)	*In vitro*	[82]
		Unknown. Polyethyleneimine (PEI) exhibits better osteogenic induction properties following engineering induction	Inoculation on 3D PLA scaffold	*In vitro*: three-dimensional scaffold structure *In vivo*: biodegradable scaffold implantation in a rat model with scraped cortical skull	[96]
	Inflammatory immunomodulation	Inhibition of LPS + IFN-*γ*-stimulated activation of M1 macrophages and induction of their conversion to M2 macrophages	None	*In vitro*	[73]
		Increases exosome content and CD73 expression, induces anti-inflammatory M2 macrophage poles, targets the Wnt5a-mediated RANKL pathway, and inhibits osteoclast activity via miR-1260b	TNF-*α* pretreated stem cell source	*In vivo*: ligature-induced periodontitis model in mice	[69]
		Reduces the expression levels of inflammatory factors TNF-*α*, IL-12, IL-1*β*, and CD86; promotes IL-10 and TNF-*α* expression levels; inhibits lipid accumulation; and promotes the polarization of pro-inflammatory macrophages into an anti-inflammatory phenotype	None	*In vitro*: lipopolysaccharide/interferon-induced inflammatory macrophages in a high-fat microenvironment	[72]
		Regulation of NF-*κ*B signaling and Wnt5a expression to reduce LPS-induced inflammatory response in periodontal stem cells	None	*In vitro*: LPS-induced inflammatory response in PDLSCs	[71]
	Skin-wound healing	Promotes collagen re-epithelialization and remodeling, angiogenesis, and neurite ingrowth	Loading chitosan/silk hydrogel sponge	*In vivo*: diabetic rat-skin defect model	[95]
		Significantly inhibits the oxidative stress-induced upregulation of HUVECs and skin fibroblasts with senescence-related genes, such as *β*-galactosidase, p21, p53, and *γ*H2AX, and mTOR/pS6 signaling pathway expression levels	None	*In vitro*	[103]
		Initiated pro-inflammatory cytokines synergistically promote anti-inflammatory M2 macrophage polarization to eliminate inflammation through the Fas/Fap-1/Cav-1 cascade response	None	*In vitro*	[70]
	Peripheral nerve regeneration	Upregulation of c-Jun N-terminal kinase, notch homolog 1, glial fibrillary acidic protein, SRY-box transcription factor 2, and other proteins activates the c-Jun N-terminal kinase-regulated repair phenotype of Schwann cells	Loading gelatin foam	*In vivo*: crush-injury mouse sciatic nerve model	[94]
		Unknown. Significantly increases the number and diameter of nerve fibers, promotes the formation of myelin sheaths, and significantly restores muscle function, nerve conduction function, and motor function	Incorporating biodegradable chitinous catheters	*In vitro*: co-culture with Chevron cells and DRGs *In vivo*: rat sciatic nerve-deficiency model	[81]
	Retinal IRI	Delivery of miR-21-5p-rich exosomes via the MEG3/miR-21-5p/PDCD4 axis	TNF-*α* pretreated stem cell source	*In vivo*: mouse vitreous model	[112]
	Tastebud regeneration	Promotes CK14 expression and regeneration of type-I, -II, and -III tastebud cells and increases BDNF and Shh expression levels and regeneration of tastebud innervation	Binding of small intestinal submucosa extracellular matrix	*In vivo*: a rat model with a critical-size tongue defect	[107]

SCAP-Exos	Inflammatory immunomodulation	Promotes Tet2-mediated Foxp3 demethylation to maintain stable Foxp3 expression and promote Treg transformation in rats	None	*In vitro*	[101]
	Dentin regeneration	Promotion of dentin salivary phosphoprotein and mineralized nodule formation in BMMSCs by differentially expressed piRNAs	None	*In vivo*: immunodeficient mice	[102]
	Regenerative root canal treatment	Promotes gene and protein expression activities of dentin salivary phosphoproteins and mineralized nodule formation and significantly increases regeneration of the dentin-pulp complex	None	*In vitro*: introduction into root fragments containing BMMSCs	[28]
	Periodontal tissue regeneration	Activation of p38 MAPK signaling pathway promotes proliferation, migration, and osteogenic differentiation of PDLSCs	None	*In vitro*	[29]
	Acute kidney injury (AKI)	LPS causes exosomes to overexpress proteins involved in antioxidant and enzyme regulatory activities, thereby inhibiting the intracellular ROS/JNK signaling pathway and promoting macrophage polarization toward the M2 phenotype	LPS pretreated stem cell source, hydrogel loaded	*In vivo*: rat model of ligature-induced periodontitis	[83]
		Inhibits the activities of SITR1, MAPK, p53, ROS, NF-*κβ*, and IL-1*β*; increases the expression of the antiapoptotic factor Bcl-2; and decreases the gene expression of the pro-apoptotic factors Bcl-2-associated X and caspase-8, CASP9, and CASP3, thereby reducing the risk of apoptosis	None	*In vitro*: cisplatin induces acute damage to rat renal epithelial cells	[75]

DFSC-Exos	Periodontal tissue regeneration	Promote the proliferation and differentiation of periodontal ligament cells from periodontitis	LPS preconditioning	*In vivo*: experimental periodontitis rat model	[83]
		Activate p38 MAPK signaling via phosphorylation to upregulate osteogenic genes (*RUNX2*, *BSP*, *COL1*) and protein expression (RUNX2, BSP, COL1, ALP)	Pretreated with ERK1/2 or p38 MAPK inhibitors	*In vitro* *In vivo*: rat periodontal defect model	[29]
		High expression of proteins involved in antioxidant and enzyme regulatory activities inhibits ROS/JNK signaling under inflammatory conditions and promotes macrophage polarization toward the M2 phenotype via ROS/ERK signaling	LPS pretreatment, loaded HA injection system	*In vitro*	[92]

## Data Availability

Data availability is not applicable to this article as no new data were created or analyzed in this study.

## Abbreviations

ESCRT: Endosomal sorting complex required for transport
MSC: Mesenchymal stem cell
DSC-Exos: Dental stem cell-derived exosomes
PDLSC-Exos: Periodontal ligament stem cell-derived exosomes
DPSC-Exos: Dental pulp stem cell-derived exosomes
SHED-Exos: Stem cells from human exfoliated deciduous teeth-derived exosomes
GMSC-Exos: Gingival mesenchymal stem cell-derived exosomes
SCAP-Exos: Stem cells from apical papilla-derived exosomes
DFSC-Exos: Dental follicle stem cell-derived exosomes
BMMSCs: Bone marrow mesenchymal stem cells
TGF-*β*: Transforming growth factor *β*
TNF: Tumor necrosis factor
FASs: First apoptosis signaling ligands
mRNA: Messenger RNA
miRNA: MicroRNA
lncRNA: Long noncoding RNA
circRNA: Circular noncoding RNA
piRNA: PIWI-interacting RNA
i-PDLSCs: Inflammatory periodontal membrane stem cells extracted from periodontitis tissues
hGMSCs: Human gingival MSCs
WNT: Wingless/integrated
MAPK: Mitogen-activated protein kinase
PI3K/Akt: Phosphoinositide 3-kinase pathway
EVs: Extracellular vesicles
ERKs: Extracellular regulated protein kinases
circLPAR1: Cyclic lysophosphatidic acid receptor 1
Th17: T helper 17 cell
IRI: Ischemia-reperfusion injury
TBI: Traumatic brain injury
OXPHOS: Oxidative phosphorylation
MMPs: Matrix metalloproteinases
IL: Interleukin
PLA: Polylactic acid
PEI: Polyethyleneimine
HUVECs: Human umbilical vein endothelial cells
LPS: Lipopolysaccharide
NF-*κ*B: Nuclear factor kappa-light-chain-enhancer of activated B cell
Bcl-2: B-cell lymphoma-2
ROS: Reactive oxygen species
JNK: Jun N-terminal kinase
Tregs: Regulatory cells
HHH: Haplotype homo
Smad: Drosophila mothers against decapentaplegic protein
STIM1: Stromal interaction molecule 1
SCI: Spinal cord injury
TLR4: Toll-like receptor 4
RANKL: Receptor activator of nuclear factor kappa-*Β* ligand.

## References

[1] Johnstone R. M., Adam M., Hammond J. R., Orr L., Turbide C. (1987). Vesicle formation during reticulocyte maturation. Association of plasma membrane activities with released vesicles (exosomes). *Journal of Biological Chemistry*.

[2] Zhang Y., Bi J., Huang J., Tang Y., Du S., Li P. (2020). Exosome: a review of its classification, isolation techniques, storage, diagnostic and targeted therapy applications. *International Journal of Nanomedicine*.

[3] Pegtel D. M., Gould S. J. (2019). Exosomes. *Annual Review of Biochemistry*.

[4] Vlassov A. V., Magdaleno S., Setterquist R., Conrad R. (2012). Exosomes: current knowledge of their composition, biological functions, and diagnostic and therapeutic potentials. *Biochimica et Biophysica Acta (BBA)—General Subjects*.

[5] Casado S., Lobo M. V. T., Paíno C. L. (2017). Dynamics of plasma membrane surface related to the release of extracellular vesicles by mesenchymal stem cells in culture. *Scientific Reports*.

[6] Gurunathan S., Kang M. H., Jeyaraj M., Qasim M., Kim J. H. (2019). Review of the isolation, characterization, biological function, and multifarious therapeutic approaches of exosomes. *Cells*.

[7] Piper R. C., Luzio J. P. (2007). Ubiquitin-dependent sorting of integral membrane proteins for degradation in lysosomes. *Current Opinion in Cell Biology*.

[8] Janockova J., Slovinska L., Harvanova D., Spakova T., Rosocha J. (2021). New therapeutic approaches of mesenchymal stem cells-derived exosomes. *Journal of Biomedical Science*.

[9] Zhang J., Li S., Li L. (2015). Exosome and exosomal microRNA: trafficking, sorting, and function. *Genomics, Proteomics & Bioinformatics*.

[10] Simons M., Raposo G. (2009). Exosomes—vesicular carriers for intercellular communication. *Current Opinion in Cell Biology*.

[11] Johnstone R. M., Bianchini A., Teng K. (1989). Reticulocyte maturation and exosome release: transferrin receptor containing exosomes shows multiple plasma membrane functions. *Blood*.

[12] Kalluri R., LeBleu V. S. (2020). The biology, function, and biomedical applications of exosomes. *Science*.

[13] Chansaenroj A., Yodmuang S., Ferreira J. N. (2021). Trends in aalivary gland tissue engineering: from stem cells to secretome and organoid bioprinting. *Tissue Engineering Part B: Reviews*.

[14] Ma S., Jiang Y., Qian Y. (2023). The emerging biological functions of exosomes from dental tissue-derived mesenchymal stem cells. *Cellular Reprogramming*.

[15] Mai Z., Chen H., Ye Y. (2021). Translational and clinical applications of dental stem cell-derived exosomes. *Frontiers in Genetics*.

[16] Gugliandolo A., Mazzon E. (2022). Dental mesenchymal stem cell secretome: an intriguing approach for neuroprotection and neuroregeneration. *International Journal of Molecular Sciences*.

[17] Chen L., Zhu S., Guo S., Tian W. (2023). Mechanisms and clinical application potential of mesenchymal stem cells-derived extracellular vesicles in periodontal regeneration. *Stem Cell Research & Therapy*.

[18] Zhao B., Chen Q., Zhao L. (2022). Periodontal ligament stem cell-derived small extracellular vesicles embedded in matrigel enhance bone repair through the adenosine receptor signaling pathway. *International Journal of Nanomedicine*.

[19] Huang C.-Y., Vesvoranan O., Yin X. (2021). Anti-inflammatory effects of conditioned medium of periodontal ligament-derived stem cells on chondrocytes, synoviocytes, and meniscus cells. *Stem Cells and Development*.

[20] Zhang Z., Shuai Y., Zhou F. (2020). PDLSCs regulate angiogenesis of periodontal ligaments via VEGF transferred by exosomes in periodontitis. *International Journal of Medical Sciences*.

[21] Gronthos S., Mankani M., Brahim J., Robey P. G., Shi S. (2000). Postnatal human dental pulp stem cells (DPSCs) in vitro and in vivo. *Proceedings of the National Academy of Sciences of the United States America*.

[22] Shoushrah S. H., Transfeld J. L., Tonk C. H. (2021). Sinking our teeth in getting dental stem cells to clinics for bone regeneration. *International Journal of Molecular Sciences*.

[23] Miura M., Gronthos S., Zhao M. (2003). SHED: stem cells from human exfoliated deciduous teeth. *Proceedings of The National Academy of Sciences of The United States of America*.

[24] Xu X., Chen C., Akiyama K. (2013). Gingivae contain neural-crest- and mesoderm-derived mesenchymal stem cells. *Journal of Dental Research*.

[25] Silvestro S., Chiricosta L., Gugliandolo A. (2020). Extracellular vesicles derived from human gingival mesenchymal stem cells: a transcriptomic analysis. *Genes*.

[26] Sonoyama W., Liu Y., Fang D. (2006). Mesenchymal stem cell-mediated functional tooth regeneration in swine. *PLoS ONE*.

[27] Xiong H., Chen K., Huang Y., Liu C. (2013). Human stem cells from apical papilla can regenerate dentin-pulp complex. *Journal of Southern Medical University*.

[28] Zhuang X., Ji L., Jiang H. (2020). Exosomes derived from stem cells from the apical papilla promote dentine-pulp complex regeneration by inducing specific dentinogenesis. *Stem Cells International*.

[29] Ma L., Rao N., Jiang H. (2022). Small extracellular vesicles from dental follicle stem cells provide biochemical cues for periodontal tissue regeneration. *Stem Cell Research & Therapy*.

[30] Hua S., Bartold P. M., Gulati K., Moran C. S., Ivanovski S., Han P. (2021). Periodontal and dental pulp cell-derived small extracellular vesicles: a review of the current status. *Nanomaterials*.

[31] Gardiner C., Di Vizio D., Sahoo S. (2016). Techniques used for the isolation and characterization of extracellular vesicles: results of a worldwide survey. *Journal of Extracellular Vesicles*.

[32] Li P., Kaslan M., Lee S. H., Yao J., Gao Z. (2017). Progress in exosome isolation techniques. *Theranostics*.

[33] Livshits M. A., Khomyakova E., Evtushenko E. G. (2015). Isolation of exosomes by differential centrifugation: theoretical analysis of a commonly used protocol. *Scientific Reports*.

[34] Morenweiser R. (2005). Downstream processing of viral vectors and vaccines. *Gene Therapy*.

[35] Colao I. L., Corteling R., Bracewell D., Wall I. (2018). Manufacturing exosomes: a promising therapeutic platform. *Trends in Molecular Medicine*.

[36] Doyle L. M., Wang M. Z. (2019). Overview of extracellular vesicles, their origin, composition, purpose, and methods for exosome isolation and analysis. *Cells*.

[37] Théry C., Amigorena S., Raposo G., Clayton A. (2006). Isolation and characterization of exosomes from cell culture supernatants and biological fluids. *Current protocols in cell biology*.

[38] Zheng Y., Dong C., Yang J. (2019). Exosomal microRNA-155-5p from PDLSCs regulated Th17/Treg balance by targeting sirtuin-1 in chronic periodontitis. *Journal of Cellular Physiology*.

[39] Cebatariuniene A., Kriauciunaite K., Prunskaite J., Tunaitis V., Pivoriunas A. (2019). Extracellular vesicles suppress basal and lipopolysaccharide-Induced NFkappaB activity in Human periodontal ligament stem cells. *Stem Cells and Development*.

[40] Liu T., Hu W., Zou X. (2020). Human periodontal ligament stem cell-derived exosomes promote bone regeneration by altering microRNA profiles. *Stem Cells International*.

[41] Fei D., Xia Y., Zhai Q. (2021). Exosomes regulate interclonal communication on osteogenic differentiation among heterogeneous osteogenic single-cell clones through PINK1/Parkin-mediated mitophagy. *Frontiers in Cell and Developmental Biology*.

[42] Lin C., Yang Y., Wang Y. (2022). Periodontal ligament fibroblasts-derived exosomes induced by PGE2 inhibit human periodontal ligament stem cells osteogenic differentiation via activating miR-34c-5p/SATB2/ERK. *Experimental Cell Research*.

[43] Dai Z., Li Z., Zheng W. (2022). Gallic acid ameliorates the inflammatory state of periodontal ligament stem cells and promotes pro-osteodifferentiation capabilities of inflammatory stem cell-derived exosomes. *Life*.

[44] Xie L., Chen J., Ren X. (2021). Alteration of circRNA and lncRNA expression profile in exosomes derived from periodontal ligament stem cells undergoing osteogenic differentiation. *Archives of Oral Biology*.

[45] Ji L., Bao L., Gu Z. (2019). Comparison of immunomodulatory properties of exosomes derived from bone marrow mesenchymal stem cells and dental pulp stem cells. *Immunologic Research*.

[46] Li B., Xian X., Lin X. (2022). Hypoxia alters the proteome profile and enhances the angiogenic potential of dental pulp stem cell-derived exosomes. *Biomolecules*.

[47] Chen Y., Ma Y., Yang X., Chen J., Yang B., Tian W. (2022). The application of pulp tissue derived-exosomes in pulp regeneration: a novel cell-homing approach. *International Journal of Nanomedicine*.

[48] Swanson W. B., Gong T., Zhang Z. (2020). Controlled release of odontogenic exosomes from a biodegradable vehicle mediates dentinogenesis as a novel biomimetic pulp capping therapy. *Journal of Controlled Release*.

[49] Vakhshiteh F., Rahmani S., Ostad S. N., Madjd Z., Dinarvand R., Atyabi F. (2021). Exosomes derived from miR-34a-overexpressing mesenchymal stem cells inhibit in vitro tumor growth: A new approach for drug delivery. *Life Sciences*.

[50] Shen Z., Kuang S., Zhang Y. (2020). Chitosan hydrogel incorporated with dental pulp stem cell-derived exosomes alleviates periodontitis in mice via a macrophage-dependent mechanism. *Bioactive Materials*.

[51] Li S., Luo L., He Y. (2021). Dental pulp stem cell-derived exosomes alleviate cerebral ischaemia-reperfusion injury through suppressing inflammatory response. *Cell Proliferation*.

[52] Liu C., Hu F., Jiao G. (2022). Dental pulp stem cell-derived exosomes suppress M1 macrophage polarization through the ROS-MAPK-NFkappaB P65 signaling pathway after spinal cord injury. *Journal of Nanobiotechnology*.

[53] Zhou Z., Zheng J., Lin D., Xu R., Chen Y., Hu X. (2022). Exosomes derived from dental pulp stem cells accelerate cutaneous wound healing by enhancing angiogenesis via the Cdc42/p38 MAPK pathway. *International Journal of Molecular Medicine*.

[54] Zhang S., Thiebes A. L., Kreimendahl F. (2020). Extracellular vesicles-loaded fibrin gel supports rapid neovascularization for dental pulp regeneration. *International Journal of Molecular Sciences*.

[55] Li J., Ju Y., Liu S., Fu Y., Zhao S. (2021). Exosomes derived from lipopolysaccharide-preconditioned human dental pulp stem cells regulate Schwann cell migration and differentiation. *Connective Tissue Research*.

[56] Xie L., Guan Z., Zhang M. (2020). Exosomal circLPAR1 promoted osteogenic differentiation of homotypic dental pulp stem cells by competitively binding to hsa-miR-31. *BioMed Research International*.

[57] W. N. Lin T., Wang L., Zhang R., Pan R., Chen Y. F. (2021). Inhibition of chondrocyte apoptosis in a rat model of osteoarthritis by exosomes derived from miR-140-5p-overexpressing human dental pulp stem cells. *International Journal of Molecular Medicine*.

[58] Jarmalaviciute A., Tunaitis V., Pivoraite U., Venalis A., Pivoriunas A. (2015). Exosomes from dental pulp stem cells rescue human dopaminergic neurons from 6-hydroxy-dopamine-induced apoptosis. *Cytotherapy*.

[59] Wang M., Li J., Ye Y., He S., Song J. (2020). SHED-derived conditioned exosomes enhance the osteogenic differentiation of PDLSCs via Wnt and BMP signaling in vitro. *Differentiation*.

[60] Pivoraite U., Jarmalaviciute A., Tunaitis V. (2015). Exosomes from human dental pulp stem cells suppress carrageenan-induced acute inflammation in mice. *Inflammation*.

[61] Luo P., Jiang C., Ji P., Wang M., Xu J. (2019). Exosomes of stem cells from human exfoliated deciduous teeth as an anti-inflammatory agent in temporomandibular joint chondrocytes via miR-*10*0-5p/mTOR. *Stem Cell Research & Therapy*.

[62] Narbute K., Pilipenko V., Pupure J. (2019). Intranasal administration of extracellular vesicles derived from human teeth stem cells improves motor symptoms and normalizes tyrosine hydroxylase expression in the substantia nigra and striatum of the 6-hydroxydopamine-treated rats. *Stem Cells Translational Medicine*.

[63] Jonavice U., Tunaitis V., Kriauciunaite K., Jarmalaviciute A., Pivoriunas A. (2019). Extracellular vesicles can act as a potent immunomodulators of human microglial cells. *Journal of Tissue Engineering and Regenerative Medicine*.

[64] Wei J., Song Y., Du Z. (2020). Exosomes derived from human exfoliated deciduous teeth ameliorate adult bone loss in mice through promoting osteogenesis. *Journal of Molecular Histology*.

[65] Huang X., Qiu W., Pan Y. (2021). Exosomes from LPS-stimulated hDPSCs activated the angiogenic potential of HUVECs in vitro. *Stem Cells International*.

[66] Huang S., Ge X., Yu J. (2017). Increased miR-124-3p in microglial exosomes following traumatic brain injury inhibits neuronal inflammation and contributes to neurite outgrowth via their transfer into neurons. *The FASEB Journal*.

[67] Wu M., Liu X., Li Z. (2021). SHED aggregate exosomes shuttled miR-26a promote angiogenesis in pulp regeneration via TGF-beta/SMAD2/3 signalling. *Cell Proliferation*.

[68] Luo L., Avery S. J., Waddington R. J. (2021). Exploring a chemotactic role for EVs from progenitor cell populations of human exfoliated deciduous teeth for promoting migration of naive BMSCs in bone repair process. *Stem Cells International*.

[69] Nakao Y., Fukuda T., Zhang Q. (2021). Exosomes from TNF-alpha-treated human gingiva-derived MSCs enhance M2 macrophage polarization and inhibit periodontal bone loss. *Acta Biomaterialia*.

[70] Kou X., Xu X., Chen C. (2018). The Fas/Fap-1/Cav-1 complex regulates IL-1RA secretion in mesenchymal stem cells to accelerate wound healing. *Science Translational Medicine*.

[71] Sun J., Wang Z., Liu P. (2022). Exosomes derived from human gingival mesenchymal stem cells attenuate the inflammatory response in periodontal ligament stem cells. *Frontiers in Chemistry*.

[72] Wang R., Ji Q., Meng C. (2020). Role of gingival mesenchymal stem cell exosomes in macrophage polarization under inflammatory conditions. *International Immunopharmacology*.

[73] Zhang Y., Wang Z., Shi B. (2021). Effect of gingival mesenchymal stem cell-derived exosomes on inflammatory macrophages in a high-lipid microenvironment. *International Immunopharmacology*.

[74] X. L. Jiang S. (2020). Exosomes from gingival mesenchymal stem cells enhance migration and osteogenic differentiation of pre-osteoblasts. *Pharmazie*.

[75] Huang T. Y., Chien M. S., Su W. T. (2022). Therapeutic potential of pretreatment with exosomes derived from stem cells from the apical papilla against cisplatin-induced acute kidney injury. *International Journal of Molecular Sciences*.

[76] Huang C.-C., Narayanan R., Alapati S., Ravindran S. (2016). Exosomes as biomimetic tools for stem cell differentiation: applications in dental pulp tissue regeneration. *Biomaterials*.

[77] Liu P., Qin L., Liu C. (2022). Exosomes derived from hypoxia-conditioned stem cells of human deciduous exfoliated teeth enhance angiogenesis via the transfer of let-7f-5p and miR-2*10*-3p. *Frontiers in Cell and Developmental Biology*.

[78] Wang M., Li J., Ye Y., Chen D., Song J. (2022). SHED-derived exosomes improve the repair capacity and osteogenesis potential of hPDLCs. *Oral Diseases*.

[79] Li Y., Yang Y. Y., Ren J. L., Xu F., Chen F. M., Li A. (2017). Exosomes secreted by stem cells from human exfoliated deciduous teeth contribute to functional recovery after traumatic brain injury by shifting microglia M1/M2 polarization in rats. *Stem Cell Research & Therapy*.

[80] Lin X., Wang H., Wu T., Zhu Y., Jiang L. (2022). Exosomes derived from stem cells from apical papilla promote angiogenesis via miR-126 under hypoxia. *Oral Diseases*.

[81] Rao F., Zhang D., Fang T. (2019). Exosomes from human gingiva-derived mesenchymal stem cells combined with biodegradable chitin conduits promote rat sciatic nerve regeneration. *Stem Cells International*.

[82] Pizzicannella J., Diomede F., Gugliandolo A. (2019). 3D printing PLA/gingival stem cells/evs upregulate miR-2861 and -210 during osteoangiogenesis commitment. *International Journal of Molecular Sciences*.

[83] Shi W., Guo S., Liu L. (2020). Small extracellular vesicles from lipopolysaccharide-preconditioned dental follicle cells promote periodontal regeneration in an inflammatory microenvironment. *ACS Biomaterials Science & Engineering*.

[84] Lan Q., Xiao X., Bi X., Gu Y., Ai Y. (2022). Effects of periodontal ligament stem cell-derived exosomes on osteoblastic proliferation, migration, differentiation, apoptosis, and signaling pathways. *Oral Diseases*.

[85] Venugopal C., K S., Rai K. S., Pinnelli V. B., Kutty B. M., Dhanushkodi A. (2018). Neuroprotection by human dental pulp mesenchymal stem cells: from billions to nano. *Current Gene Therapy*.

[86] Ti D., Hao H., Tong C. (2015). LPS-preconditioned mesenchymal stromal cells modify macrophage polarization for resolution of chronic inflammation via exosome-shuttled let-7b. *Journal of Translational Medicine*.

[87] Huang H.-M., Han C.-S., Cui S.-J. (2022). Mechanical force-promoted osteoclastic differentiation via periodontal ligament stem cell exosomal protein ANXA3. *Stem Cell Reports*.

[88] Zheng J., Kong Y., Hu X. (2020). MicroRNA-enriched small extracellular vesicles possess odonto-immunomodulatory properties for modulating the immune response of macrophages and promoting odontogenesis. *Stem Cell Research & Therapy*.

[89] Shimizu Y., Takeda-Kawaguchi T., Kuroda I. (2022). Exosomes from dental pulp cells attenuate bone loss in mouse experimental periodontitis. *Journal of Periodontal Research*.

[90] Hu X., Zhong Y., Kong Y., Chen Y., Feng J., Zheng J. (2019). Lineage-specific exosomes promote the odontogenic differentiation of human dental pulp stem cells (DPSCs) through TGFbeta1/smads signaling pathway via transfer of microRNAs. *Stem Cell Research & Therapy*.

[91] Guo H., Li B., Wu M. (2021). Odontogenesis-related developmental microenvironment facilitates deciduous dental pulp stem cell aggregates to revitalize an avulsed tooth. *Biomaterials*.

[92] Huang Y., Liu Q., Liu L., Huo F., Guo S., Tian W. (2022). Lipopolysaccharide-preconditioned dental follicle stem cells derived small extracellular vesicles treating periodontitis via reactive oxygen species/mitogen-activated protein kinase signaling-mediated antioxidant effect. *International Journal of Nanomedicine*.

[93] Zhou H., Li X., Yin Y. (2020). The proangiogenic effects of extracellular vesicles secreted by dental pulp stem cells derived from periodontally compromised teeth. *Stem Cell Research & Therapy*.

[94] Mao Q., Nguyen P. D., Shanti R. M. (2019). Gingiva-derived mesenchymal stem cell-extracellular vesicles activate schwann cell repair phenotype and promote nerve regeneration. *Tissue Engineering Part A*.

[95] Shi Q., Qian Z., Liu D. (2017). GMSC-derived exosomes combined with a chitosan/silk hydrogel sponge accelerates wound healing in a diabetic rat skin defect model. *Frontiers in Physiology*.

[96] Diomede F., Gugliandolo A., Cardelli P. (2018). Three-dimensional printed PLA scaffold and human gingival stem cell-derived extracellular vesicles: a new tool for bone defect repair. *Stem Cell Research & Therapy*.

[97] Yu D., Li Y., Wang M. (2022). Exosomes as a new frontier of cancer liquid biopsy. *Molecular Cancer*.

[98] Moshy S. E., Radwan I. A., Rady D. (2020). Dental stem cell-derived secretome/conditioned medium: the future for regenerative therapeutic applications. *Stem Cells International*.

[99] Ivica A., Ghayor C., Zehnder M., Valdec S., Weber F. E. (2020). Pulp-derived exosomes in a fibrin-based regenerative root filling material. *Journal of Clinical Medicine*.

[100] Chen Y., Koshy R., Guirado E., George A. (2021). STIM1 a calcium sensor promotes the assembly of an ECM that contains extracellular vesicles and factors that modulate mineralization. *Acta Biomaterialia*.

[101] Yu S., Chen X., Liu Y. (2022). Exosomes derived from stem cells from the apical papilla alleviate inflammation in rat pulpitis by upregulating regulatory T cells. *International Endodontic Journal*.

[102] Wang A., Liu J., Zhuang X. (2020). Identification and comparison of piRNA expression profiles of exosomes derived from human stem cells from the apical papilla and bone marrow mesenchymal stem cells. *Stem Cells and Development*.

[103] Shi H. Z., Zeng J. C., Shi S. H., Giannakopoulos H., Zhang Q. Z., Le A. D. (2021). Extracellular vesicles of GMSCs alleviate aging-related cell senescence. *Journal of Dental Research*.

[104] Xu X.-Y., Tian B.-M., Xia Y. (2020). Exosomes derived from P2X7 receptor gene-modified cells rescue inflammation-compromised periodontal ligament stem cells from dysfunction. *Stem Cells Translational Medicine*.

[105] Lei F., Li M., Lin T., Zhou H., Wang F., Su X. (2022). Treatment of inflammatory bone loss in periodontitis by stem cell-derived exosomes. *Acta Biomaterialia*.

[106] Tibensky M., Jakubechova J., Altanerova U. (2022). Gene-directed enzyme/prodrug therapy of rat brain tumor mediated by human mesenchymal stem cell suicide gene extracellular vesicles in vitro and in vivo. *Cancers*.

[107] Zhang Y., Shi S., Xu Q., Zhang Q., Shanti R. M., Le A. D. (2019). SIS-ECM laden with GMSC-derived exosomes promote taste bud regeneration. *Journal of Dental Research*.

[108] Gurunathan S., Kang M.-H., Kim J.-H. (2021). A comprehensive review on factors influences biogenesis, functions, therapeutic and clinical implications of exosomes. *International Journal of Nanomedicine*.

[109] Li M., Li S., Du C. (2020). Exosomes from different cells: characteristics, modifications, and therapeutic applications. *European Journal of Medicinal Chemistry*.

[110] He C., Zheng S., Luo Y., Wang B. (2018). Exosome theranostics: biology and translational medicine. *Theranostics*.

[111] Guo J., Zhou F., Liu Z. (2022). Exosome-shuttled mitochondrial transcription factor A mRNA promotes the osteogenesis of dental pulp stem cells through mitochondrial oxidative phosphorylation activation. *Cell Proliferation*.

[112] Yu Z., Wen Y., Jiang N. (2022). TNF-alpha stimulation enhances the neuroprotective effects of gingival MSCs derived exosomes in retinal ischemia-reperfusion injury via the MEG3/miR-21a-5p axis. *Biomaterials*.

[113] Ana I. D., Barlian A., Hidajah A. C., Wijaya C. H., Notobroto H. B., Wungu T. D. K. (2021). Challenges and strategy in treatment with exosomes for cell-free-based tissue engineering in dentistry. *Future Science OA*.

[114] Chiricosta L., Silvestro S., Gugliandolo A. (2020). Extracellular vesicles of human periodontal ligament stem cells contain microRNAs associated to proto-oncogenes: implications in cytokinesis. *Frontiers in Genetics*.

[115] Wang Z., Maruyama K., Sakisaka Y. (2019). Cyclic stretch force induces periodontal ligament cells to secrete exosomes that suppress IL-1*β* production through the inhibition of the NF-*κ*B signaling pathway in macrophages. *Frontiers in Immunology*.

[116] Liu R., Dong M., Han W., Dong J., Niu W. (2023). Application and progress of small extracellular vesicles in periodontal and pulp regenerat. *Chinese Journal of Tissue Engineering Research*.

[117] Stanko P., Altanerova U., Jakubechova J., Repiska V., Altaner C. (2018). Dental mesenchymal stem/stromal cells and their exosomes. *Stem Cells International*.

[118] Zhou H., Li X., Yin Y. (2022). Correction: the proangiogenic effects of extracellular vesicles secreted by dental pulp stem cells derived from periodontally compromised teeth. *Stem Cell Research & Therapy*.

[119] Liu Y., Zhuang X., Yu S. (2021). Exosomes derived from stem cells from apical papilla promote craniofacial soft tissue regeneration by enhancing Cdc42-mediated vascularization. *Stem Cell Research & Therapy*.

[120] Alcayaga-Miranda F., Varas-Godoy M., Khoury M. (2016). Harnessing the angiogenic potential of stem cell-derived exosomes for vascular regeneration. *Stem Cells International*.

[121] van der Meel R., Sulheim E., Shi Y., Kiessling F., Mulder W. J. M., Lammers T. (2019). Smart cancer nanomedicine. *Nature Nanotechnology*.

[122] Konala V. B. R., Bhonde R., Pal R. (2020). Secretome studies of mesenchymal stromal cells (MSCs) isolated from three tissue sources reveal subtle differences in potency. *In Vitro Cellular & Developmental Biology—Animal*.

